# Inferences from structural comparison: flexibility, secondary structure wobble and sequence alignment optimization

**DOI:** 10.1186/1471-2105-13-S15-S12

**Published:** 2012-09-11

**Authors:** Gaihua Zhang, Zhen Su

**Affiliations:** 1 State Key Laboratory of Plant Physiology and Biochemistry, College of Biological Sciences, China Agricultural University, Beijing 100094, People's Republic of China

## Abstract

**Background:**

Work on protein structure prediction is very useful in biological research. To evaluate their accuracy, experimental protein structures or their derived data are used as the 'gold standard'. However, as proteins are dynamic molecular machines with structural flexibility such a standard may be unreliable.

**Results:**

To investigate the influence of the structure flexibility, we analysed 3,652 protein structures of 137 unique sequences from 24 protein families. The results showed that (1) the three-dimensional (3D) protein structures were not rigid: the root-mean-square deviation (RMSD) of the backbone C_α _of structures with identical sequences was relatively large, with the average of the maximum RMSD from each of the 137 sequences being 1.06 Å; (2) the derived data of the 3D structure was not constant, e.g. the highest ratio of the secondary structure wobble site was 60.69%, with the sequence alignments from structural comparisons of two proteins in the same family sometimes being completely different.

**Conclusion:**

Proteins may have several stable conformations and the data derived from resolved structures as a 'gold standard' should be optimized before being utilized as criteria to evaluate the prediction methods, e.g. sequence alignment from structural comparison. Helix/β-sheet transition exists in normal free proteins. The coil ratio of the 3D structure could affect its resolution as determined by X-ray crystallography.

## Background

The best way to investigate the functions and mechanism of proteins at the molecular level is to obtain their three-dimensional (3D) structures [1-3]. However, it is time-consuming and expensive to determine protein structures by experimental methods and this has meant that resolved protein structures have lagged greatly behind known protein sequences [2,4]. Scientists have spent decades on protein structure prediction to accelerate the process of obtaining protein structures. To advance the progress of protein structure prediction, Critical Assessment of protein Structure Prediction (CASP) experiments have highlighted the shortcomings in this field [1,5]. In general, the experimentally resolved protein structures, especially structures resolved by X-ray crystallography, and their derived data are used as the criteria to evaluate the accuracy of methods of protein structure prediction [1,6]. For example, to assess the predicted 3D structures, structural comparisons were performed between resolved structures and their predicted models, and root-mean-square deviation (RMSD) [7], TM-score [8], HBscore [1,9], GDT-HA or GDT-TS [1,5,7] were used to evaluate the difference.

In fact, thermodynamics and kinetics dictate that protein structures are not static [10]. Work on enzyme catalytic mechanisms indicate that there are diverse steady conformations for a single enzyme and they could cooperatively change [11]. In addition, previous works has shown that even under the same crystallization conditions, protein structures have marked variations [12,13]. Thus the structure determined by X-ray crystallography may be one of many conformations of a protein, and so it is inadequate to evaluate predicted models with limited experimental structures. Additionally, as proteins are dynamic machines [14,15] we can infer that their derived data should also not be unique. Secondary structure wobble has demonstrated that the secondary structure can change and that there are limits to evaluation of protein prediction accuracy [16,17].

In the present study, some redundant data deposited in PDB http://www.rcsb.org/[18] were collected to investigate the characters of protein flexibility and evaluate its influence on criteria for the assessment of work related to structure prediction. At the 3D structural level, the maximum RMSD of backbone C_α _of two structures with identical sequences could reach 5.43Å. At the secondary structural level, we found helix/β-sheet transitions in normal free proteins which had only been reported previously in prion or protein complexes [17,19-21]. Furthermore, with increasing resolution value, the ratio of the coil state in secondary structure increased. At primary structural level, the sequence alignments from structural comparisons are variable in that there may be wrongly aligned sites in the datasets [22] that are used as criteria in the computational methods of sequence alignment. Then with analysis of the characters of sequence alignments from structural comparison [e.g. secondary structure, evolutionary distance (ED) and gaps] some suggestions for sequence alignment optimization were proposed.

## Materials and methods

### Data collection

CD-HIT [23] was utilized for clustering the protein sequences from the PDB database [18], the sequence identity threshold used was 0.99 as we tried to analyse the structures with few mutations, because these mutated sites are in or around the functional important region that have often been altered by researchers in mechanisms studies. HMMER3 was utilized to categorize the protein family with an E-value cut-off of 0.0001 [24]. The structures were selected using the following rules:

1. The sequential structures were determined by X-ray crystallography with resolution < 3.5Å;

2. There were > 4 structures for each identical sequence;

3. In each protein family, there were at least three unique proteins.

In general, structures with resolution < 2.5 Å are considered reliable. However, analysis of structures with low resolution may supply some interesting information about protein flexibility. In the present study, 1,956 PDB entries were collected, with 1,588 having resolution < 2.5 Å (Additional file 1: Figure S1 and Additional file 2).

Structures with identical sequences were defined as a 'structural group'. We obtained 3,652 structures from 137 unique sequences and distributed in 24 protein families; and 62 structural groups contained mutations. The detailed protein families can be seen in Additional file 3; the PDB entries and mutation sites are shown in Additional file 4. The structural folding types were annotated by the SCOP 1.75 database [25] and shown in Additional file 5. The functional divisions are shown in Additional file 6. The dataset includes free proteins, protein-ligand complexes and protein-protein complexes.

### The flexibility of the protein structure

To analyse the flexibility of the 3D structure, TM-align [8] was utilized for structural comparisons. There were 88,036 structural comparisons obtained within the same structural group, which were utilized to indicate the flexibility of the 3D structure. There were 284,599 structural comparisons obtained from comparisons between structural groups within the same protein family, which were utilized to analyse the sequence alignment variation.

### Secondary structure wobble

DSSP [26] was utilized to calculate the secondary structure in investigation the secondary structure wobble. Then the secondary structures were translated into three states: for 'E' to 'E', indicating β-sheet; for 'H', 'I' and 'G' to 'H', indicating helix; and the others were to 'C', indicating coil. We aligned all sequences derived from structures in a group using MUSCLE [22] to examine the secondary structure states of the equivalent site. If one site had more than one secondary structure state, it was called a secondary structure wobble [16].

The wobble sites ratio was calculated using equation 1. The 'Wobble Total' was defined as the ratio of all wobble sites in a structural group. The 'Wobble Single' was defined as the ratio of all wobble sites in two compared structures.

To show that flexibility is a character of the proteins, we selected structures for wobble analysis based on many different requirements, e.g. without any different ligands, ions or other molecules.

(1) $$R_{w}=N_{w}/N_{a}\times 100\%$$

*R_w _*is the wobble sites ratio, *N_w _*is the number of the wobble site and *N_a _*is the total number of protein sites.

### Relationships between resolution and secondary structure wobble

The resolution of the structures may also be determined by their flexibility. Here, we investigated the relationships between resolution and wobble ratio. In brief, if there was a wobble site between two structures, the secondary structure states were added to the equivalent certain resolution values (the gradient value is 0.1 Å). After that the ratio of the coil state under a certain resolution set was calculated for all structures. Then the Pearson's correlation coefficient (PCC) between the resolution and the ratio of coil state was calculated. Finally, a linear relationship between resolution and coil ratio was found. In addition, we checked the wobble ratio of structures with similar resolution.

### Structural comparison and sequence alignment variation

Here, we defined 'group pairs' as the results of the structural comparison of two structural groups. The 'group pairs' within the same family were utilized for sequence alignment variation analysis. If a site aligned the same residues in all sequence alignments from group pairs, it was defined as a 'common site'; or else defined as a 'multi-site'. If a site aligned a gap in all the comparisons, it was defined as a "gap site". We used equation 2 to calculate the ratio to reveal the sequence alignment variation.

(2) $$R_{x}=N_{x}/N_{a}\times 100\%$$

*N_a _*is the average of the two proteins' length; *N_x _*is the number of common sites (*N_c_*) or multi-sites (*N_m_*) or gap sites (*N_g_*); and *R_x _*is the ratio of *N_x _*to *N_a_*, *R_c _*corresponds to *N_c_*, *R_m _*to *N_m_*, and *R_g _*to *N_g_*.

### Sequence alignment and secondary structure

The sequence alignment based on structural comparison was not unique, so that we tried to optimize them. The secondary structure is usually used to help the sequence alignment and so we calculated the ratio of the secondary structure states of the three alignment states (common, gap and multi sites) for each family. For the wobble sites, if there were two secondary structure states in one site, then we added 0.5 to equivalent secondary structure state number. Finally, we calculated the average of these ratios.

### ED comparison

In theory, high structural similarity corresponds to low ED. RMSD and TM-score were utilized to measure the structural similarity. In each of the group pairs, two pairs of sequence alignments were selected based on the maximum and minimum of RMSD and TM-score. Equation 3 was utilized for ED calculation between the aligned sequences *S_x_* and *S_y _*which contain ***n ***aligned sites [27].

(3) $$ED(S_{x}S_{y})=[1-\frac{2\times \overset{n}{\underset{i}{\sum }}M_{S_{xi}S_{yi}}}{\overset{n}{\underset{i}{\sum }}M_{S_{xi}S_{xi}}+\overset{n}{\underset{i}{\sum }}M_{S_{yi}S_{yi}}}]\times 100$$

$M_{S_{xi}S_{yi}}$ is the score of the *i^th ^*aligned residues pairs in *S_x _*and *S_y _*followed the score matrix BLOSUM62. $M_{S_{xi}S_{xi}}$ and $M_{S_{yi}S_{yi}}$ are similar to $M_{S_{xi}S_{yi}}$, but with the *i^th ^*site pairs of *S_x _*or the *i^th ^*site pairs of *S_y_*, respectively. *ED (S_x_S_y_) *is the ED of sequences *S_x _*and *S_y_*.

### Gaps of the exceptions in the ED comparison

For the above ED comparison, some pairs' sequence alignments were not consistent with the hypothesis. Therefore, we further analysed the gaps difference of these exceptions. Firstly, the residues without aligned residues on both ends of the sequence alignment were deleted. Secondly, the number of gap-opening and gap-extension were counted. Thirdly, we compared the number of gaps of these exceptions in the ED comparison.

### Statistical analysis

In this study, all statistical analyses were carried out using the statistical package R [28]. The PCC analysis and classical regression were done with the cor.test and lm function respectively. Chi-square tests for calculation of significant differences were done using the chisq.test function.

## Results and discussion

### Protein structure flexibility

**Figure 1:**
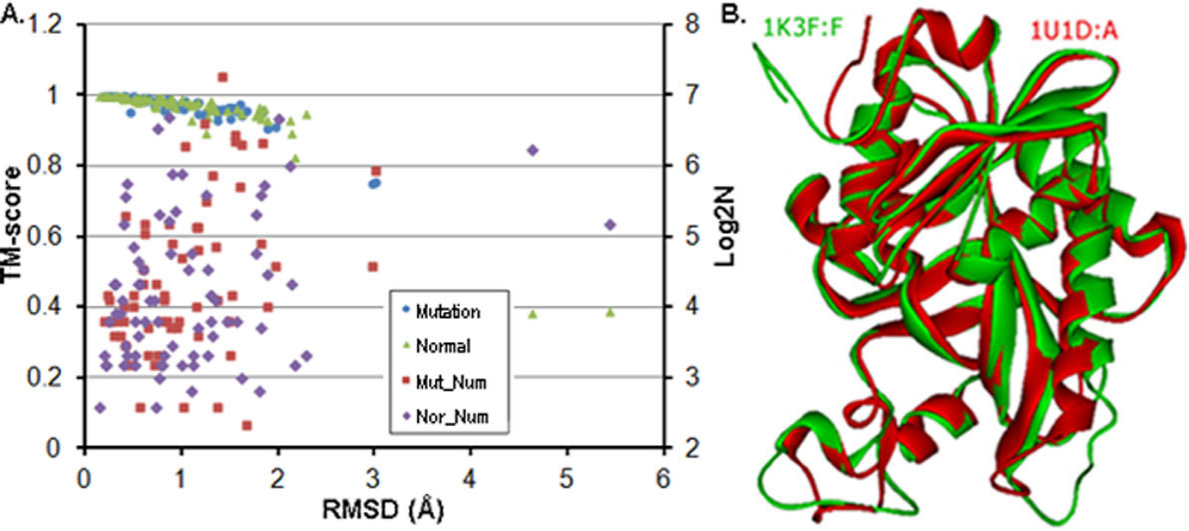
**Protein structural flexibility**. (**A**) The maximum RMSD and equivalent minimum TM-score of the structural groups. The red rectangles and purple diamonds represent the base 2 logs of the structural numbers in structural groups with (blue dots) or without mutations (green triangles), respectively. (**B**) Structural comparison of PDB: 1U1D: A (red) [31] and PDB: 1K3F: F (green) [32].

Protein structures are flexible [14,15]. The maximum RMSDs and the equivalent minimum TM-scores within the structural groups are shown in Figure 1A. The maximum RMSD was 5.43Å (2BCX: A [29] and 2IX7: B [30]) and most of their equivalent residues were not at the same position. The average of the maximum RMSD of the 137 groups was 1.06 Å; for the 62 structural groups with mutations the average of the maximum RMSD was 1.03 Å, while for the remaining 75 structural groups was 1.08 Å. Combining the RMSD distributions of the groups with or without mutations, showed that the few mutations had little effect on global 3D structure. The scale of the structural groups can also affect the RMSD and TM-score (Figure 1A). In addition, ions, ligands and other proteins could cause more structural changes (data not shown).

Two structures with identical sequence (PF01048) are compared in Figure 1B; the structural changes between the regular secondary structural segments could lead the structures to be clearly different to each other. The 3D topological structures were still conserved.

Except the impact of extrinsic factors, proteins are intrinsic not static, even when arrayed in a crystal [13]. The process to obtain structural data by X-ray crystallography would determine that the protein molecule is arrayed in an orderly pattern in the crystal for signal amplification and enhancement. The structural data from the experiment may be the last conformation before the protein crystal was froze in liquid nitrogen; however, at room temperature, the protein may transform from one conformation to another. Thus if we assess predicted models by structural comparison with a limited number of resolved structures, the result may be unreliable. Since the crystallization conditions of the resolved structures were known, we could use these parameters in molecular dynamic (MD) simulation and collect conformations with high RMSD but little energy difference to build a structural set as criteria.

### Secondary structure wobble

**Figure 2:**
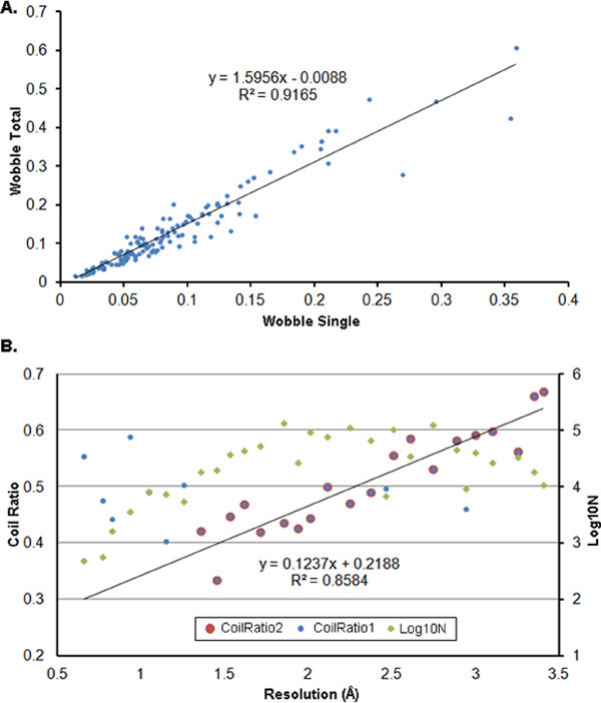
**Secondary structure wobble**. (A) Relationship between 'wobble total' and 'wobble single'. There are about 37.5% ((1.5956 - 1)/1.5956) wobble sites only exist in the structures which not including the two structures related to 'Wobble Single'. (B) The relationship between resolution and secondary structure wobble. Green diamonds represent the base-10 logs of the numbers of the wobble site pairs. The blue dots indicate the ratio of coil sites in wobble sites under certain resolution bins of size 0.1 Å, and the red dots indicate the selected blue dots with > 10,000 wobble sites.

Secondary structure wobble is a result of structural flexibility. The maximum wobble site ratio was 60.69% (Figure 2A). Helix/β-sheet transitions were found (Table 1 and Additional file 7: Figure S2) which were not previously reported in normal free proteins [17]; however, this was a small probability event.

**Table 1:** Types and frequency of secondary structural wobbles

	Total	Wobbles	Ratio (%)	C<=>E	C<=>H	H<=>E
Sites Num	33,899	4,027	12.00	1,273	2,736	18
Mutation	412	72	17.48	23	49	0

'Sites Num' is the number of residue sites in the 137 proteins; 'Mutation' is the number of sites contains mutations. 'C<=>E', 'C<=>H' and 'H<=>E' are coil/β-sheet, coil/helix and helix/β-sheet transitions, respectively.

We further found a strong linear relationship between the 'Wobble Total' and the maximum 'Wobble Single' (Figure 2A). The two structures of the maximum 'Wobble Single' could be considered as two extremely different conformations in inactive or active states for the protein to perform its function. That is, about 37.5% of wobble sites only appeared in the intermediate conformations of protein (Figure 2A), and were thus considered essential for the proteins to perform their function. In addition, this indicates that the wobble sites or residues of proteins may move with each other in a coordinated and continuous pattern.

In addition, the ratios of wobble sites in protein-protein complexes were higher than in free proteins (see Additional file 8: Figure S3). However, differences of the ratios of wobble sites are not clearly between proteins with or without ligands/ions (see Additional file 9: Figure S4).

The results above indicate that it is insufficient to utilize the derived secondary structure as criteria to directly evaluate methods of secondary structure prediction. However, we can also employ MD simulation to generate a secondary structure dataset as criteria. Furthermore, if we use structures as training sets for works on structural prediction, we should construct a set as comprehensive as possible, otherwise, much useful information may be lost, especially in the highly flexibility zone. For example, HYPROSP II [33], a knowledge-based secondary structure prediction method, performed best as it utilized comprehensive data training for prediction.

### Mutational sites and wobble sites

Some structures contain few mutations, and we calculated the wobble sites ratio in these mutational sites and compared it to total sites (Table 1). The mutational sites contained relatively high wobble site ratios. The Chi-square test indicated a significant difference between them (X^2 ^= 11.59, P < 0.01). In addition, most of the original residues of these mutation sites were wobbles. Therefore, the sites in or around the functionally important regions should be of higher flexibility as noted by previous studies [17].

### Relationships between resolution and secondary structure wobble

With decreasing resolution, the coil site ratio increased (Figure 2B). The analysis indicates the number of coil sites in a structure could affect its resolution according to X-ray crystallography. In addition, with decreasing resolution, the wobble sites ratio increased (Additional file 10: Figure S5).

### Sequence alignment variations

**Figure 3:**
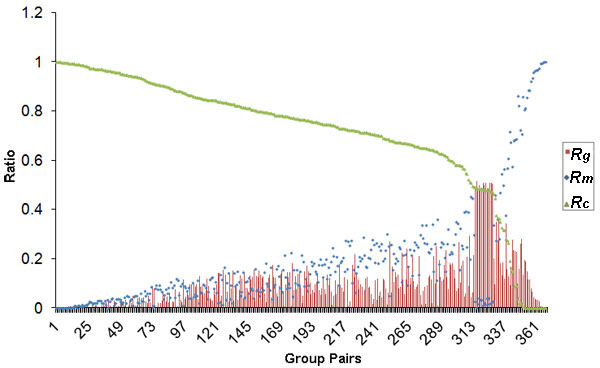
**Sequence alignment variations**. The average of the common sites ratio (*R_c_*) was 71.36%, the multi-sites ratio (*R_m_*) was 18.78% and the gap sites ratio (*R_g_*) was 9.86%. The horizontal axis indicates the compared group pairs.

There were 368 group pairs generated from structural comparisons among the structural groups in the same protein family. The sequence alignments from the structural comparison were not constant (Figure 3) when some group pairs had no common site. There was a relatively high positive correlation between RMSD_max _and *R_m _*(PCC = 0.78, P < 2.20 × 10^-16^). Therefore, with increasing structural difference, sequence alignment from structural comparison would be less reliable.

### Sequence alignment and secondary structure

**Figure 4:**
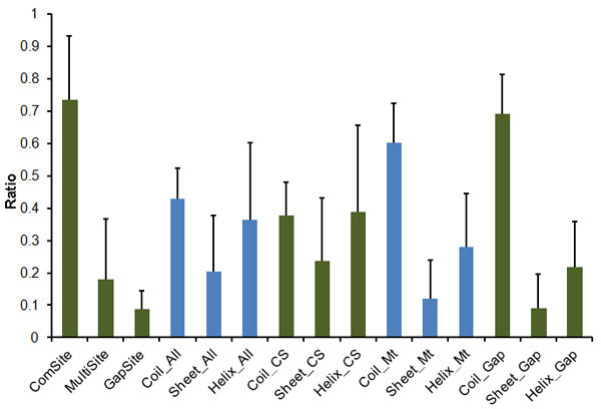
**Sequence alignment and secondary structure**. ComSite, GapSite and MultiSite: the average of the common sites, gap sites and multi-sites ratios of each family, respectively. Coil_All, Helix_All and Sheet_All: the average of the ratio of the coil state, helix state and sheet state of each family, respectively. Coil_CS, Helix_CS and Sheet_CS: the average of the ratio of the coil state, helix state and sheet state of each family in the zone of common sites, respectively. Coil_Mt, Helix_Mt and Sheet_Mt: the average of the ratio of the coil state, helix state and sheet state of each family in the zone of multi-sites, respectively. Coil_Gap, Helix_Gap and Sheet_Gap: the average of the ratio of the coil state, helix state and sheet state of each family in the zone of gap sites, respectively.

There were 181,733 residues used in the study of secondary structure states distribution in the sequence alignment. The ratio of coil state was high in the zone of multi-sites and gap sites (Figure 4). The chi-square test showed the difference was significant between coil to helix and coil to β-sheet (data not shown). This indicates that the residues in coil state are more flexible and we could refine the sequence alignment of segments enriched in coil state.

### ED, gaps and sequence alignment optimization

**Figure 5:**
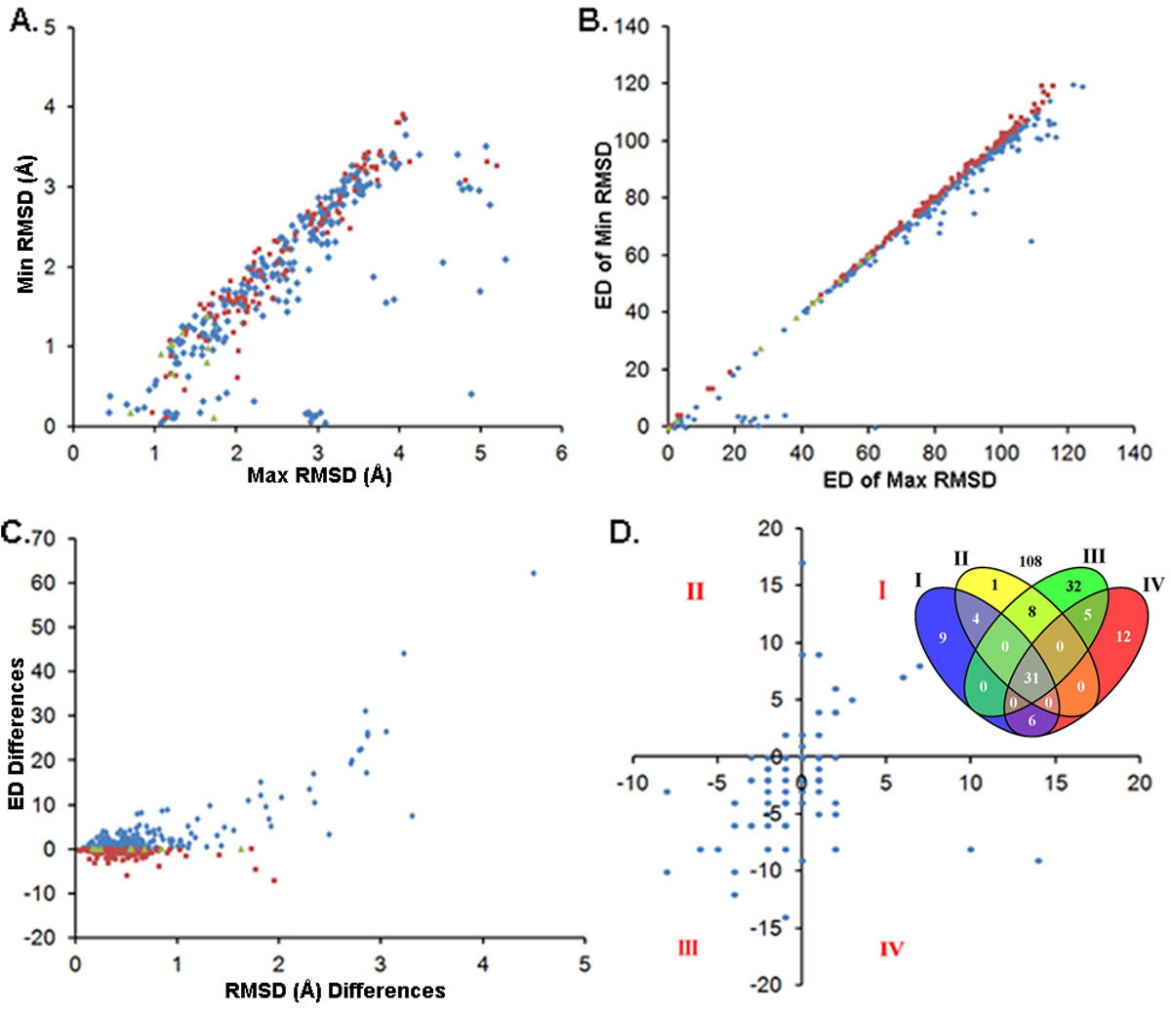
**RMSD and ED**. The samples obeying the theoretical hypothesis are shown in blue diamonds, the exceptions are shown in red rectangles and where the ED difference was zero is shown in green triangles (A, B and C). (A) The distribution of the minimum/maximum RMSD. (B) The distribution of the ED of corresponding RMSD. (C) X-axis: differences of the maximum RMSD subtract the equivalent minimum RMSD; Y-axis: differences of the ED of the minimum RMSD subtract the ED of the maximum RMSD. (D) Gap difference of the exceptions. X-axis (gap-openings) and Y-axis (gap-extensions) are the differences of the gaps of the minimum RMSD subtract the maximum RMSD, respectively. The dots in quadrant IV indicate the gap-openings of the minimum RMSD are higher than the maximum RMSD, but the gap-extensions are reverse. The Venn diagram shows the dot number in quadrants I, II, III and IV, and on the axes and the origin e.g. there are 31 dots at the origin, 32 dots in quadrant of III and 12 dots in quadrant of IV.

There were 368 pairs sequence alignments for comparison analysis based on RMSD. In theory, the lower the RMSD, the lower was the ED. The distribution of maximum/minimum RMSDs and EDs are shown in Figure 5A and 5B, respectively. However, there were 108 pairs that did not follow this rule. The difference between RMSDs and equivalent EDs are shown in Figure 5C. Compared to the samples obeying the theoretical hypothesis, the ED difference of the exceptions were smaller. For the exceptions, the difference in the number of gap-openings and gap-extensions is shown in Figure 5D, this shows that most of the sequence alignments with minimum RMSD had less gaps, and especially gap-extensions.

At the same time, we analysed 368 pairs of sequence alignments, selected based on TM-score, and obtained similar results. Theoretically speaking, the higher the TM-score, the lower was the ED. However, there were 153 pairs that did not follow the rule (see Additional file 11: Figure S6).

The analysis of sequence alignment indicated that sequence alignment based on structural comparison would not be the best. Proteins are not static and the residues adjacent in 3D space may move relative to each other along the sequence. Therefore, the sequence alignment should reflect the dynamic movement of proteins. That means that aligned residues should have similar dynamic characters.

The analysis of ED, gaps and the distributions of the gap sites and multi-sites indicates that sequence alignment from structural comparison could be optimized, based on substitution score matrix, especially in regions with coil state. There are many software packages that could complete this job.

Additionally, RMSD measured by software was not accurate enough to reveal the difference between structures. There was a strong positive correlation between minimum RMSD and its ED (PCC = 0.92, P < 0.01); however, it was worse between maximum RMSD and its ED (PCC = 0.76, P < 0.01) (see Additional file 12: Figure S7). The comparison indicated that the minimum RMSD was closer to the native RMSD; and so the sequence alignment of the minimum RMSD may be more credible. In addition, we may be able to construct a quantified relationship between the RMSD of 3D structures and the ED from their sequence alignment.

## Conclusions

Native proteins are not static, as stored in the PDB database, because they must perform their functions in a dynamic pattern. In addition, experimental errors and other extrinsic factors could cause structural changes. In the present study, the main protein folding types were collected for flexibility analysis (Additional file 5). We conclude that not only enzymes, but also other proteins, may have many stable conformations and could cooperatively change. Therefore, if we want to evaluate the accuracy of methods of structural prediction, we may need to employ MD simulation to construct a structure set as criteria. For sequence alignments from structural comparison, we could also optimize the segments enriched in coil states using existing software packages for sequence alignment based on score matrix. Compared to other residues, the residues in or around the active region are more flexible. The fact that a higher coil ratio could reduce resolution may encourage scientists working on experimental protein structure to determine methods to decrease the coil ratio in protein and thus improve their resolution.

## Competing interests

The authors declare that they have no competing interests.

## Authors' contributions

GZ analysed the data and drafted the manuscript. ZS supervised this study.

## Supplementary Material

Additional file 1 **Figure S1: The numbers of selected PDB entries with resolution with a gradient value of 0.1 Å**. Click here for file

Additional file 2 **PDB entries and their resolution**. Some PDB entries have two resolution values. Click here for file

Additional file 3 **Selected protein families**. Click here for file

Additional file 4 **The selected PDB entries, their sequences and mutational sites**. Click here for file

Additional file 5 **SCOP class of the Pfam ID**. Click here for file

Additional file 6 **Functional divisions of selected protein families**. Click here for file

Additional file 7 **Figure S2: Helix/β-sheet transition**. Three pair protein structures are shown, with existing helix/β-sheet transitions and the equivalent zone marked yellow. (**A**) 1DSE: A and 2AS3: A, PF00141, 69Y, 70R; (**B**) 1AIG: M and 1PSS: M, PF00124, 26A, 27N; (**C**) 1GJM: A and 1T87: B, 82R, 83E, 86E, 87A. Besides these structure pairs, there are a total of nine families of helix/β-sheet transitions: PF00061, PF00124 and PF00139 are not enzymes; PF00067, PF00141 and PF00186 are enzymes with coenzymes and PF00215, PF00561 and PF01048 are enzymes without coenzymes. Click here for file

Additional file 8 **Figure S3: The wobble ratios of free proteins (A) and protein-protein complexes (B)**. Of the 137 structural groups, 64 were free proteins, and 73 contained protein-protein complexes. Click here for file

Additional file 9 **Figure S4: The wobble ratios of the structures without ligands (A) and with the same ligands (B)**. Of the 137 structural groups, 30 contained some structures without ligands, and 111 contained some structural pairs with the same ligands. Then their wobble ratios were counted. Click here for file

Additional file 10 **Figure S5: Resolution and wobble ratio**. The structures were classified into six datasets based on their resolution value at a gradient value of 0.5 Å. Then the wobble ratio was calculated and the number of proteins in each dataset was marked on the histogram. Click here for file

Additional file 11 **Figure S6: TM-score and ED**. The samples obeying the hypothesis are shown in blue diamonds, the exceptions are shown in red rectangles and where the ED difference was zero is shown in green triangles (A, B and C). (**A**) The distribution of the minimum/maximum TM-score. (**B**) The distribution of the ED of corresponding TM-score. (**C**) X-axis: differences of the maximum TM-score subtract the equivalent minimum TM-score; Y-axis: differences of the ED of the maximum TM-score subtract the ED of the minimum TM-score. (**D**) Gap difference of the exceptions. X-axis (gap-openings) and Y-axis (gap-extensions) are the differences of the gaps of the maximum TM-score subtract the minimum TM-score, respectively. The dots in quadrant IV indicate the gap-openings of the maximum TM-score are higher than the minimum TM-score, but the gap-extensions are reverse. The Venn diagram shows the dot number in quadrants I, II, III and IV, and on the axes and the origin e.g. there are 39 dots at the origin. Click here for file

Additional file 12 **Figure S7: RMSD and ED**. Click here for file
