# Supplementary figures and images for: Inferences from structural comparison: flexibility, secondary structure wobble and sequence alignment optimization
Source: BMC Bioinformatics. 2012 Sep 11;13(Suppl 15):S12. doi: 10.1186/1471-2105-13-S15-S12 (PMC3439719; doi:10.1186/1471-2105-13-S15-S12)

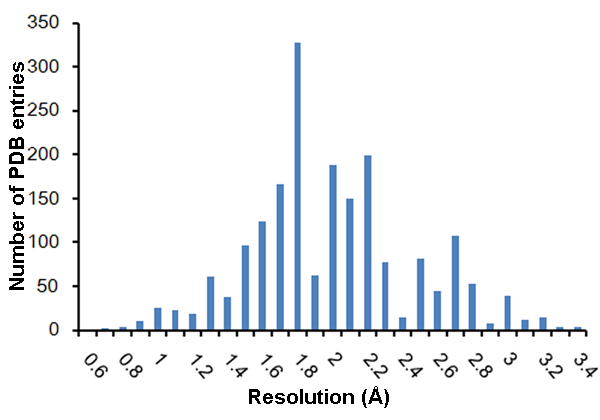

Supplement: Additional file 1 — Figure S1: The numbers of selected PDB entries with resolution with a gradient value of 0.1 Å. [file 1471-2105-13-S15-S12-S1.tif]

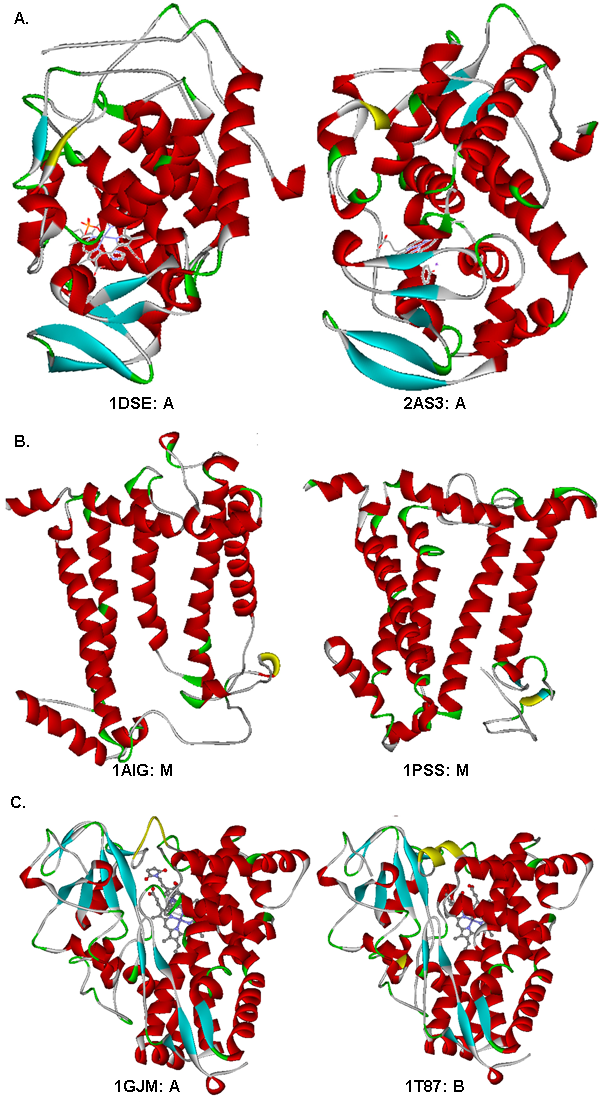

Supplement: Additional file 7 — Figure S2: Helix/β-sheet transition. Three pair protein structures are shown, with existing helix/β-sheet transitions and the equivalent zone marked yellow. (A) 1DSE: A and 2AS3: A, PF00141, 69Y, 70R; (B) 1AIG: M and 1PSS: M, PF00124, 26A, 27N; (C) 1GJM: A and 1T87: B, 82R, 83E, 86E, 87A. Besides these structure pairs, there are a total of nine families of helix/β-sheet transitions: PF00061, PF00124 and PF00139 are not enzymes; PF00067, PF00141 and PF00186 are enzymes with coenzymes and PF00215, PF00561 and PF01048 are enzymes without coenzymes. [file 1471-2105-13-S15-S12-S7.tif]

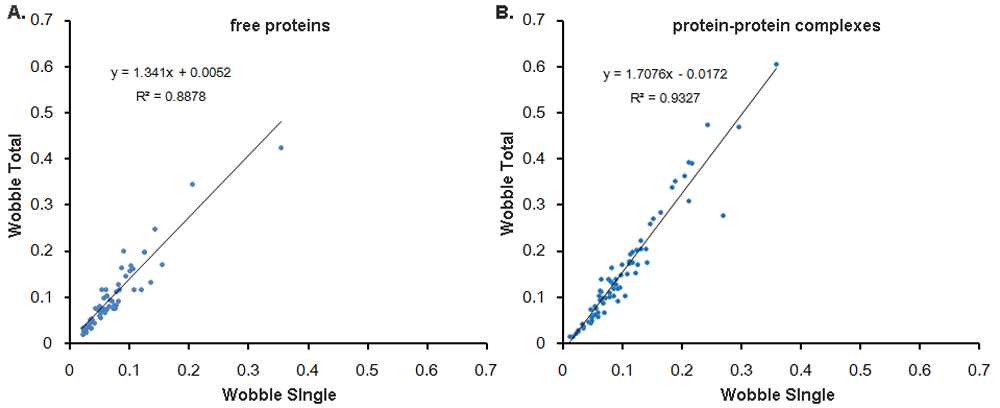

Supplement: Additional file 8 — Figure S3: The wobble ratios of free proteins (A) and protein-protein complexes (B). Of the 137 structural groups, 64 were free proteins, and 73 contained protein-protein complexes. [file 1471-2105-13-S15-S12-S8.tif]

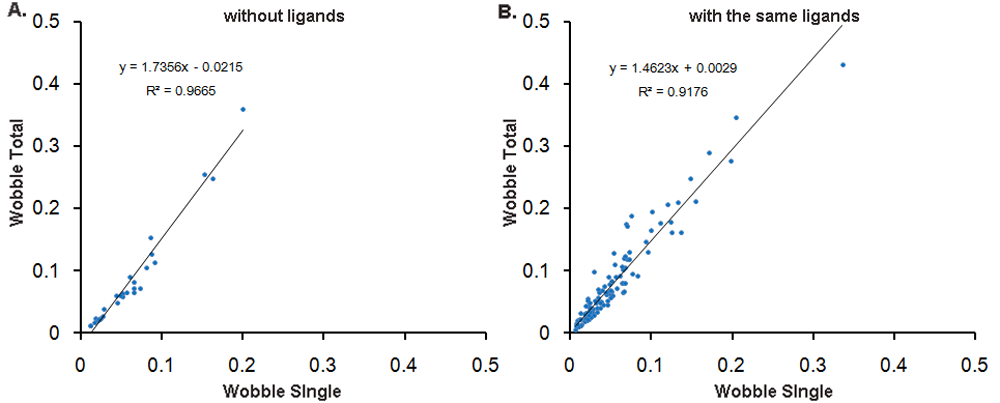

Supplement: Additional file 9 — Figure S4: The wobble ratios of the structures without ligands (A) and with the same ligands (B). Of the 137 structural groups, 30 contained some structures without ligands, and 111 contained some structural pairs with the same ligands. Then their wobble ratios were counted. [file 1471-2105-13-S15-S12-S9.tif]

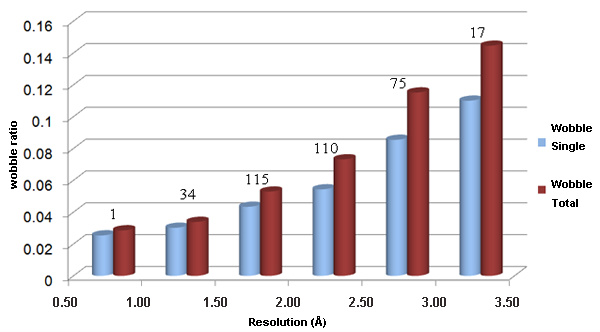

Supplement: Additional file 10 — Figure S5: Resolution and wobble ratio. The structures were classified into six datasets based on their resolution value at a gradient value of 0.5 Å. Then the wobble ratio was calculated and the number of proteins in each dataset was marked on the histogram. [file 1471-2105-13-S15-S12-S10.tif]

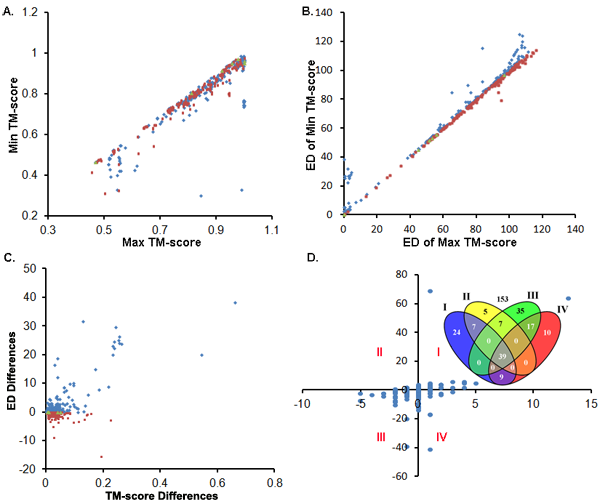

Supplement: Additional file 11 — Figure S6: TM-score and ED. The samples obeying the hypothesis are shown in blue diamonds, the exceptions are shown in red rectangles and where the ED difference was zero is shown in green triangles (A, B and C). (A) The distribution of the minimum/maximum TM-score. (B) The distribution of the ED of corresponding TM-score. (C) X-axis: differences of the maximum TM-score subtract the equivalent minimum TM-score; Y-axis: differences of the ED of the maximum TM-score subtract the ED of the minimum TM-score. (D) Gap difference of the exceptions. X-axis (gap-openings) and Y-axis (gap-extensions) are the differences of the gaps of the maximum TM-score subtract the minimum TM-score, respectively. The dots in quadrant IV indicate the gap-openings of the maximum TM-score are higher than the minimum TM-score, but the gap-extensions are reverse. The Venn diagram shows the dot number in quadrants I, II, III and IV, and on the axes and the origin e.g. there are 39 dots at the origin. [file 1471-2105-13-S15-S12-S11.tif]

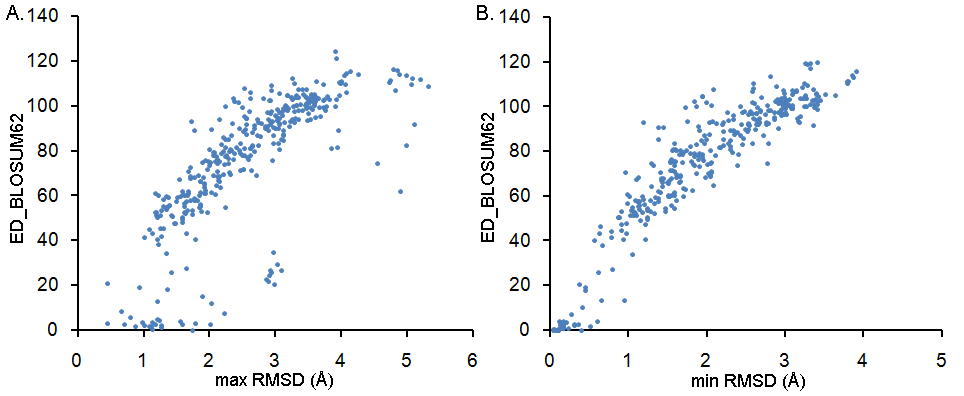

Supplement: Additional file 12 — Figure S7: RMSD and ED. [file 1471-2105-13-S15-S12-S12.tif]
