# Supplementary material for: Inferences from structural comparison: flexibility, secondary structure wobble and sequence alignment optimization
Source: BMC Bioinformatics. 2012 Sep 11;13(Suppl 15):S12. doi: 10.1186/1471-2105-13-S15-S12 (PMC3439719; doi:10.1186/1471-2105-13-S15-S12)
Supplement: Additional file 3 — Selected protein families. [file 1471-2105-13-S15-S12-S3.doc]

### Additional file 3 - Selected Protein families

| **Pfam ID** | **Num** | **Pfam ID** | **Num** | **Pfam ID** | **Num** | **Pfam ID** | **Num** |
| --- | --- | --- | --- | --- | --- | --- | --- |
| PF00026 | 3 | PF00127 | 6 | PF00124 | 4 | PF00248 | 7 |
| PF00215 | 3 | PF00210 | 6 | PF00348 | 4 | PF00067 | 8 |
| PF00233 | 3 | PF00232 | 6 | PF00036 | 5 | PF00561 | 8 |
| PF07686 | 3 | PF00959 | 6 | PF00073 | 5 | PF00061 | 9 |
| PF00104 | 4 | PF00141 | 7 | PF00337 | 5 | PF00139 | 9 |
| PF00121 | 4 | PF00186 | 7 | PF00080 | 6 | PF01048 | 9 |
| ‘Pfam ID’: protein families. ‘Num’: the number of the unique proteins. There are 137 unique sequences, 1,955 different PDB IDs and 3,652 independent structures (see ‘Additional file 4’). | | | | | | | |
