# Supplementary material for: Inferences from structural comparison: flexibility, secondary structure wobble and sequence alignment optimization
Source: BMC Bioinformatics. 2012 Sep 11;13(Suppl 15):S12. doi: 10.1186/1471-2105-13-S15-S12 (PMC3439719; doi:10.1186/1471-2105-13-S15-S12)
Supplement: Additional file 5 — SCOP class of the Pfam ID. [file 1471-2105-13-S15-S12-S5.doc]

## Additional file 5 –SCOP class of the Pfam ID

| **SCOP Class** | **Pfam ID** |
| --- | --- |
| a | PF00036, PF00067, PF00104, PF00141, PF00210, PF00233, PF00348 |
| b | PF00026, PF00061, PF00080, PF00127, PF00139, PF00337, PF07686, PF00073 |
| c | PF00121, PF00186, PF00215, PF00232, PF00248, PF00561, PF01048 |
| d | PF00959 |
| f | PF00124 |
