# Supplementary material for: Inferences from structural comparison: flexibility, secondary structure wobble and sequence alignment optimization
Source: BMC Bioinformatics. 2012 Sep 11;13(Suppl 15):S12. doi: 10.1186/1471-2105-13-S15-S12 (PMC3439719; doi:10.1186/1471-2105-13-S15-S12)
Supplement: Additional file 6 — Functional divisions of selected protein families. [file 1471-2105-13-S15-S12-S6.doc]

## Additional file 6 –Functional divisions of selected protein families

In the 24 protein families, eight were not enzymes; 12 and four enzymes were without and with coenzymes, respectively. As the collected protein families have great structural diversity they could represent most structural types. In addition, many structural groups contained protein-protein complexes.

**Enzymes:** PF00026, PF00067, PF00080, PF00121, PF00141, PF00186, PF00215, PF00232, PF00233, PF00248, PF00337, PF00348, PF00561, PF00959 and PF01048.

**With coenzyme** (the coenzymes’ abbreviations in the structure data are shown after the protein families’ accession number, some of them may be analogues of the real coenzyme):

PF00067: HEM, ZNH and MNR;

PF00141: HEM, PP9, ZNH, FMI, CCH, TBV, DDH and TBV;

PF00248: NAP, NDP and NAD;

PF00186: NAP, NDP, DZF, FOL, TQD, DTM, CO4, MTX, MXA, DHF, FFO and DDF.

**Without coenzyme**: PF00026, PF00080, PF00121, PF00215, PF00232, PF00233, PF00337, PF00348, PF00561, PF00959 and PF01048.

**Non-enzyme**: PF00036, PF00061, PF00073, PF00104, PF00124, PF00127, PF00139, PF00210 and PF07686.
